# A study of antimicrobial activity, acute toxicity and cytoprotective effect of a polyherbal extract in a rat ethanol-HCl gastric ulcer model

**DOI:** 10.1186/1756-0500-5-546

**Published:** 2012-10-02

**Authors:** Emmanuel E Haule, Mainen J Moshi, Ramadhani SO Nondo, Dennis T Mwangomo, Rogasian LA Mahunnah

**Affiliations:** 1 Department of Biological and Preclinical Studies, Institute of Traditional Medicine, Muhimbili University of Health and Allied Sciences, P.O. Box 65001, Dar es Salaam, Tanzania; 2 Department of Medical Botany, Plant Breeding and Agronomy, Institute of Traditional Medicine, Muhimbili University of Health and Allied Sciences, P.O. Box 65001, Dar es Salaam, Tanzania

**Keywords:** Ozoroa insignis, Maytenus senegalensis, Entada abyssinica, Lannea schimperi, Gastroprotection, Toxicity

## Abstract

**Background:**

The decoction of the aerial parts of *Rhynchosia recinosa* (A.Rich.) Bak. [Fabaceae] is used in combination with the stem barks of *Ozoroa insignis* Del. (Anacardiaceae), *Maytenus senegalensis* (Lam.) Excell. [Celastraceae] *Entada abyssinica* Steud. ex A.Rich [Fabaceae] and *Lannea schimperi* (Hochst.)Engl. [Anacardiaceae] as a traditional remedy for managing peptic ulcers. However, the safety and efficacy of this polyherbal preparation has not been evaluated. This study reports on the phytochemical profile and some biological activities of the individual plant extracts and a combination of extracts of the five plants.

**Methods:**

A mixture of 80% ethanol extracts of *R. recinosa, O. insignis, M. senegalensis, E. abyssinica* and *L. schimperi* at doses of 100, 200, 400 and 800 mg/kg body wt were evaluated for ability to protect Sprague Dawley rats from gastric ulceration by an ethanol-HCl mixture. Cytoprotective effect was assessed by comparison with a negative control group given 1% tween 80 in normal saline and a positive control group given 40 mg/kg body wt pantoprazole. The individual extracts and their combinations were also tested for antibacterial activity against four Gram negative bacteria; *Escherichia coli* (ATCC 25922), *Salmonella typhi* (NCTC 8385), *Vibrio cholerae* (clinical isolate), and *Klebsiella pneumoniae* (clinical isolate) using the microdilution method. In addition the extracts were evaluated for brine shrimp toxicity and acute toxicity in mice. Phytochemical tests were done using standard methods to determine the presence of tannins, saponins, steroids, cardiac glycosides, flavonoids, alkaloids and terpenoids in the individual plant extracts and in the mixed extract of the five plants.

**Results:**

The combined ethanolic extracts of the 5 plants caused a dose-dependent protection against ethanol/HCl induced ulceration of rat gastric mucosa, reaching 81.7% mean protection as compared to 87.5% protection by 40 mg/kg body wt pantoprazole. Both the individual plant extracts and the mixed extracts of 5 plants exhibited weak to moderate antibacterial activity against four G-ve bacteria. Despite *Ozoroa insignis* being toxic to mice at doses above 1000 mg/kg body wt, the other plant extracts and the combined extract of the 5 plants were tolerated by mice up to 5000 mg/kg body wt. The brine shrimp test results showed the same pattern of toxicity with *Ozoroa insignis* being the most toxic (LC_50_ = 10.63 μg/ml). Phytochemical tests showed that the combined extract of the five plants contained tannins, saponins, steroids, cardiac glycosides, flavonoids and terpenoids. Flavonoids, tannins and terpenoids are known to have antioxidant activity.

**Conclusion:**

The combined extract of the five plants exhibited a dose-dependent protective activity in the rat ethanol-HCl gastric ulcer model. The extracts also exhibited weak antibacterial activity against four Gram negative bacteria and low acute toxicity in mice and brine shrimps. Although the results support claims by traditional healers who use a decoction of the five plants for treatment of peptic ulcers, more models of gastric ulceration and proper animal toxicity studies are needed to validate possible clinical use of the polyherbal extract. It is also evident that the doses of the crude extracts showing protection of the gastric mucosa are too large for realistic translation to direct clinical application, but further studies using bioassay guided fractionation are important to either identify more practical fractions or active compound/s.

## Background

The Haya people of Kagera region are endowed with a culture rich in traditional medicine practice owed to an extensive intercultural exchange among the diverse ethnic tribes of the Lake Victoria Basin [1]. In this series of reports combined documentation [1,2] and evaluation to establish proof of the concept is being done. In previous reports a good corroboration of claims on antimicrobial activity has been established from laboratory tests [3-5] and using evidence from the literature which has indicated an average 20-30% agreement with the therapeutic claims by the Kagera traditional healers [1,2].

One of the plants used in traditional medicine is *Rhynchosia recinosa* (A.Rich.) Bak. (Fabaceae) [Akashakanyaliyoya]. The aerial parts of this plant are pounded together with the stem barks of *Ozoroa insignis* Del. (Anacardiaceae) [Omkerenge]*, Maytenus senegalensis* (Lam.) Excell. (Celastraceae) [Omurosho], *Entada abyssinica* Steud. ex A. Rich(Fabaceae) [Mwiganjula] and *Lannea schimperi* (Hochst.)Engl. (Anacardiaceae) [Omusangesange] and then boiled with water to make a decoction. The decoction is administered at a dose of one glass three times a day for the treatment of peptic ulcers. In this study a mixture of extracts of the five plants was evaluated for ability to offer protection against ethanol-HCl induced gastric ulceration [6-8], antibacterial activity against some Gram negative bacteria, acute toxicity in mice and brine shrimp toxicity.

## Methods

### Materials

Ethanol (absolute) was bought from Fluka Chemie GmbH (Sigma-Aldrich®, Zwijndrecht, Netherlands) while dimethyl sulfoxide (DMSO) was purchased from Sigma® (Poole, Dorset, UK). Tryptone Soya broth was bought from HIMEDIA® (Himedia Laboratories Pvt Ltd, Mumbai, INDIA. *Escherichia coli* (ATCC 25922), *Salmonella typhi* (NCTC 8385), *Vibrio cholerae* (clinical isolate), and *Klebsiella pneumoniae* (clinical isolate) were obtained from the Department of Microbiology and Immunology, Muhimbili University of Health and Allied Sciences (MUHAS). *p-*Iodonitrotetrazolium chloride (INT) was bought from SIGMA® (Sigma- Aldrich®, St Louis, USA), while pantoprazole (lyophilized powder for i.v injection; Batch No. JKJ 3534D and manufactured by Sun Pharmaceutical Industries Ltd, Halol-Baroda Highway, Halol-389350, Gujarat, India.), was bought from a local pharmacy. The brine shrimp eggs were purchased from Aquaculture Innovations (Grahamstown 6140, South Africa) and sea salt was prepared locally by evaporating water collected from the Indian Ocean, along the Dar es Salaam Coast. Cyclophosphamide, NEOPHOS 500® (CIPLA Ltd, MIDC Boisar, INDIA) was purchased from a local Pharmacy in Dar es Salaam, Tanzania.

### Collection of plant material

The plants were collected and identified by Mr. Selemani Haji of the Department of Botany University of Dar es Salaam and the voucher specimen; *R. resinosa* (MJM 3506)*, O. insignis* (MJM 3476), *E. abyssinica* (MJM 3466)*, L. schimperi* (MJM 3411) and *M. senegalensis* (MJM 3428) are kept in the Herbaria of the Institute of Traditional Medicine, MUHAS and Department of Botany University of Dar es Salaam.

### Preparation of plant extracts

All the plant samples were air-dried and ground into powder using a plant milling machine. Samples of each plant material were macerated with 80% ethanol at room temperature for 24h and filtered through Whatman No.1 filter paper. This procedure was repeated three times to ensure complete extraction of the plant material. The extracts were concentrated by evaporation under reduced pressure in a rotary evaporator at 40°C and residual water removed by freeze drying. The percentage extract yields obtained were *Rhynchosia resinosa* (14.4%), *Ozoroa insignis* (2.0%), *Entada abyssinica* (5.3%), *Maytenus senegalensis* (2.2%) and *Lannea schimperi* (5.9%).

### Phytochemical screening

The different extracts were tested for the presence of steroids, saponins, flavonoids, terpenoids, cardiac glycosides, alkaloids and tannins using standard methods [9,10].

### Preparation and administration of the polyherbal extract

A mixture of 80% ethanol extracts of *R. recinosa* (**the main plant of the recipe**), *O. insignis, M. senegalensis, E. abyssinica* and *L. schimperi* at doses of 100, 200, 400 and 800 mg/kg body wt were evaluated for ability to protect Sprague Dawley rats from gastric ulceration by an ethanol-HCl mixture. Cytoprotective effect was assessed by comparison with a negative control group given 1% tween 80 in normal saline and a positive control group given 40 mg/kg body wt pantoprazole. Equal amounts of 80% ethanol extracts of each of the five plants were mixed together to obtain a uniform mixture. The mixture was dissolved in 1% tween 80 to make 40, 80 and 160 mg/ml solutions. Rats were given 5 ml/kg body wt of these solutions, which translates to 200, 400 and 800 mg/kg body wt, respectively.

### Induction of peptic ulcers

Both male and female Sprague Dawley rats weighing 100-188g were used. The rats were fasted for 36 h but allowed free access to drinking water [11]. Thirty rats were randomly allocated to 5 groups of 6 rats each. The rats in group one (Solvent control) were orally pre-dosed with 5 ml/kg body wt 1% tween 80 in normal saline; rats in group two were orally pre-dosed with 40 mg/kg body wt pantoprazole as positive control [12], and rats in groups 3, 4 and 5 were pre-dosed with 200, 400 and 800 mg/kg body wt solution of the combined extracts one hour before oral administration of 5 ml/kg body wt of the ulcerogenic mixture. The ulcerogenic mixture contained 80% ethanol and 5% hydrochloric acid in distilled water [6-8]. The animals were euthanized using ether anaesthesia 4h after the ulcerogenic dose of the ethanol/HCl mixture. The stomachs were removed, cut open along the greater curvature, washed with normal saline and observed for the severity of ulcers [13]. Each stomach was examined grossly and the degree of ulceration was graded according to the method of Shay et al.[14] with some modifications as follows: 0 = no lesions (normal stomach); 0.5 = hyperemia (red coloration); 1 = hemorrhagic spots, 2 = 1–5 small ulcers; 3 = many small ulcers, 4 = many small and large ulcers; 6 = stomach full of ulcers along with perforations. Percentage protection was calculated by comparison with the untreated control group [14].

Percentage protection

(1) $$=100-\frac{Mean\;ulcer\;index\;of\;treated\;group}{Mean\;ulcer\;index\;of\;control\;group}\times 100$$

### Statistical analysis

The ulcer index results are expressed as mean ± standard deviation (SD). The results of ulcer index were analyzed using Kruskal-Wallis one way analysis of variance. The means were compared using non-parametric Kruskal-Wallis test.

### Testing for antibacterial activity

Four Gram-negative bacteria *Escherichia coli* (ATCC 25922)*, Salmonella typhi* (NCTC 8385)*,* and clinical isolates of *Vibrio cholerae* and *Klebsiella pneumoniae* were used for the study. Minimum inhibitory concentrations (MICs) were determined using the microdilution method [15]. Stock solutions of the extracts were prepared by dissolving 100 mg of extracts into 1 ml of DMSO (100mg/ml). Each of the 96 well microtitre plates were first preloaded with 100 μl of the broth media followed by addition of 100 μl of the extracts into the first well of each row to make a total volume of 200μl in the first wells. After thorough mixing 100 μl were drawn from each of the first row wells and put into the next row wells. The process was repeated down the columns to the last wells at the bottom where 100 μl was discarded. Thereafter, 100μl of the bacterial suspensions (0.5Mac Farland standard turbidity) were added to each well to make a final volume of 200 μl in each well. Gentamicin sulphate (100μg/ml) was used as the standard drug. Rows containing broth, DMSO and bacteria were included as negative controls (solvent control) and rows containing broth and bacteria only were included in order to see if there was bacterial growth or not (growth control). The plates were then incubated at 37°C for 24h. After incubation for 24 h, at 37°C, 40μl of 0.02% *p*-iodonitrotetrazolium (INT) chloride solution was added to each well followed by incubation for 1h at 37°C. Bacterial growth was indicated by a change in color to pink in the wells. Absence of bacterial growth was indicated by no color change of the dye. The first concentration at which no bacterial growth occurred was taken as the MIC.

### Brine shrimps lethality test

The brine shrimp lethality test (BST) was used to predict the presence, in the extracts, of cytotoxic activity [16]. Stock solutions of the extracts were made in DMSO and then adjusted to 5ml with artificial sea to obtain concentrations ranging from 8–240 μg/ml and incubated in duplicate vials with brine shrimp larvae. Cyclophosphamide which was a positive control for cytotoxic activity was dissolved in distilled water. Ten brine shrimp larvae were placed in each of the duplicate vials. Control brine shrimp larvae were placed in a mixture of artificial sea water (3.8 g/l sea salt) and DMSO only. DMSO concentrations were restricted to 0.6% or below. After 24 h the nauplii were examined against a lighted background, and the average number of survived larvae in each duplicate vial was determined.

### Data analysis

The mean results of brine shrimp mortality were plotted against the logarithms of concentrations using the Fig P computer program (Biosoft Inc, USA), which also gives the regression equations. The regression equations were used to calculate LC_16_, LC_50_ and LC_84_ values. Confidence intervals (95% CI) were then calculated using the three results [17]. An LC_50_ value greater than 100 μg/ml was considered to represent an inactive extract.

### Acute toxicity studies

Both male and female Theiller’s original albino mice were used. The mice were acclimatized in an air conditioned room at 20°C for 7 days before experimentation. Before dosing with extracts the mice were starved for 36 h but allowed access to adequate drinking water, and kept in cages with wire mesh bottoms to prevent coprophagy. Initially a dose of 1000 mg/kg body wt was administered to a group of 6 mice (3 male and 3 female), and the mice observed for signs of immediate toxicity and/or death for 72 h. If no toxicity was observed another group of 3 male and 3 female mice was given a dose of 2000 mg/kg body wt and the same observations made. If no signs of toxicity or death occurred doses were sequentially increased to 3000, 4000, and 5000 mg/kg body wt, respectively. Extracts were solubilized in 1% tween 80 and administered at a single oral dose volume of 5 ml/kg body wt or two separate 5 ml/kg body wt doses given within an hourly interval, depending on solubility. A control group was run for each plant extract which was administered a single 5 ml or two 5 ml/kg body wt of 1% tween 80 to match with the volume of plant extract administered.

### Ethical clearance

This study was given ethical clearance by the Muhimbili University of Health and Allied Sciences Ethical Review Board.

## Results

### Phytochemical screening

The results of phytochemical tests as reported in Table 1 show that tannins were present in all the 5 plant extracts and in the mixture of the 5 plants, except in the *R.recinosa* extract. Steroids and terpenoids were present in all the 5 plant extracts and the mixed extract of the 5 plants. Alkaloids were only detected in the *O. insignis* extract but not the other 4 plants or the mixture. Flavonoids were present in *L. schimperi, O. insignis, E. abyssinica* extracts and the mixed extract of the 5 plants. The other compounds are as reported in Table 1.

**Table 1:** Phytochemical compounds present in plant extracts

**Extract**	**Tannins**	**Saponins**	**Steroids**	**Cardiac glycosides**	**Flavonoids**	**Alkaloids**	**Terpenoids**
*L. schimperi*	+	-	+	+	+	-	+
*R. resinosa*	-	+	+	-	-	-	+
*O. insignis*	+	+	+	+	+	+	+
*M. senegalensis*	+	+	+	+	-	-	+
*E. abssynica*	+	-	+	+	+	-	+
mixture	+	+	+	+	+	-	+

**Key:** + = means indicated compounds are present; - means indicated compounds are absent.

### Antiulcerogenic activity

**Figure 1:**
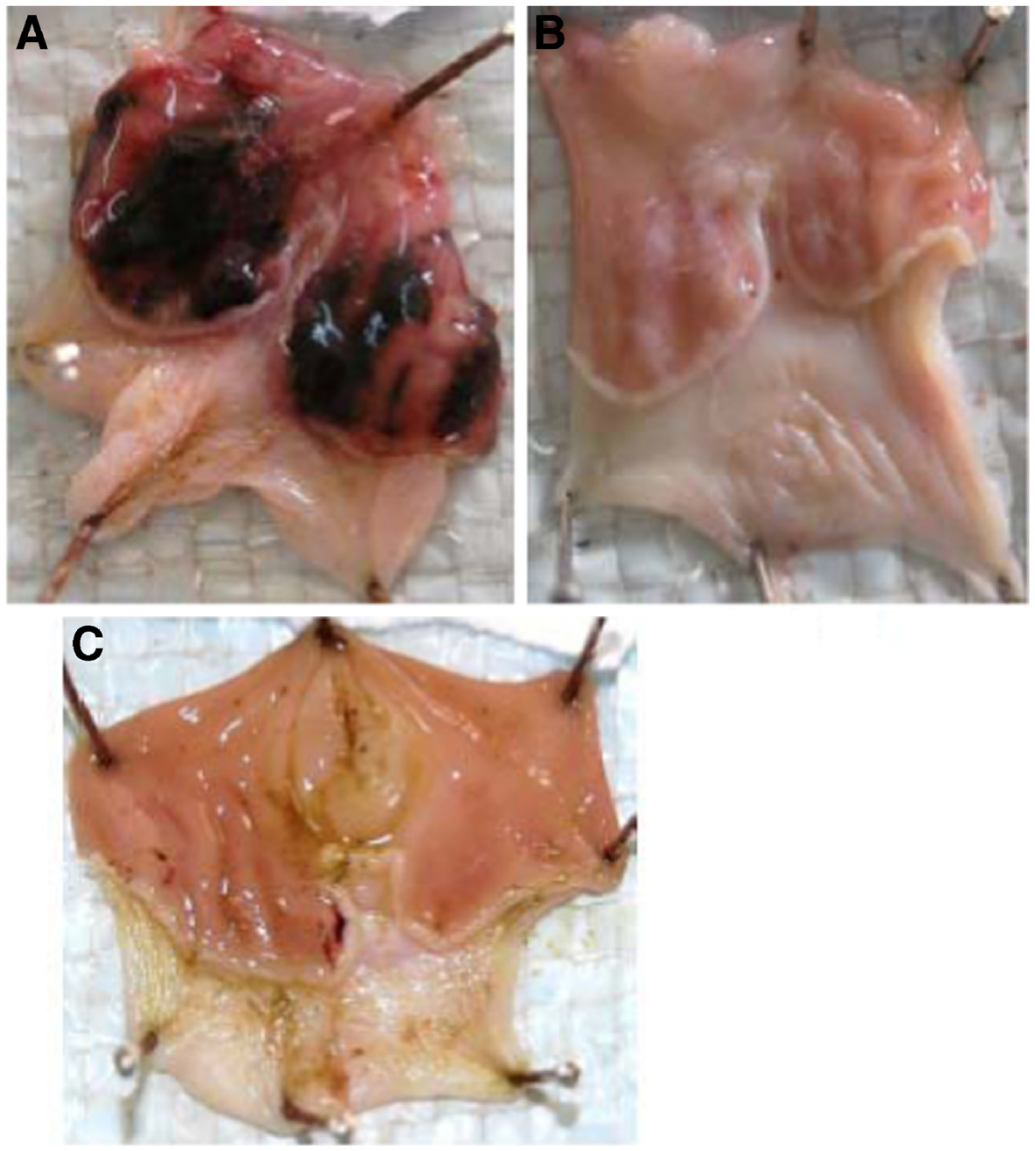
**Photographs showing the gross appearance of the rat stomachs following insult with ethanol/HCl (80%/5%) mixture. **In **A **the rat was pre-dosed with the solvent before insult with ethanol/HCl mixture. In **B **the rat was pre-dosed with 40 mg/kg body wt pantoprazole before exposure to the ulcerative mixture and in **C **the rat was predosed with 800 mg/kg body wt of the combined extracts of the 5 plants. Dark areas or spots indicate the ulcerated spots.

The combined 80% ethanolic extracts of *O. insignis, E. abyssinica, L. schimperi, R. recinosa* and *M. senegalensis* protected rat gastric mucosa from ulceration by the ethanol-HCl mixture. Figure 1 shows photographs of the gross appearance of rat stomachs pre-treated with 1% tween 80 in normal saline, 800 mg/kg body wt of a mixture of the 5 plant extracts, and 40 mg/kg body wt pantoprazole, respectively. The mixed extract exhibited a dose-dependent gastro-protective activity, which increased from 28.3% at a dose of 200 mg/kg body wt (P ≤ 0.001) to 53.3 (P ≤ 0.001) at 400 mg/kg body wt and 81.7% (P ≤ 0.001) at 800 mg/kg body wt, as compared to solvent treated rats (Table 2). Pantoprazole, a proton pump inhibitor, at 40 mg/kg body wt, achieved 87.5% mean gastroprotection (P ≤ 0.001). All the three doses and pantoprazole showed a significantly lower ulcer index as compared to the solvent treated rats (P ≤ 0.001). There was a significant difference in ulcer index scores between the 3 extract doses used, and between 40 mg/kg body wt pantoprazole and both 200 and 400 mg/kg body wt of the extract (P ≤ 0.001). However at the dose of 800 mg/kg body wt the mean ulcer index score of the extract was comparable to that of 40 mg/kg body wt pantoprazole (P > 0.05), indicating that at this dose the extract was as good as pantoprazole.

**Table 2:** Effect of combined ethanolic extracts on ethanol/HCl-induced gastric ulcers in the rat

**Treatment**	**Dose (mg/kg body wt)**	**Mean ulcer index (n = 6)**	**%ge Protection**
**Solvent control**	0	6.0	0
**Combined 5 plant extracts**	200 mg/kg	4.3 ± 0.8	28.3*
**Combined 5 plant extracts**	400 mg/kg	2.8 ±1.2	53.3*
**Combined 5 plant extracts**	800 mg/kg	1.1 ± 0.8	81.7*
**Pantoprazole**	40 mg/kg	0.7 ± 0.9	87.5*

Each value is a mean of results of 6 rats. * = significant P ≤ 0.001 compared to solvent controls.

### Antibacterial activity

The aqueous ethanol extracts of all the five plants exhibited some antibacterial activity with MICs ranging from 0.8-12.5 mg/ml (Table 3). The highest activity was shown by *M. senegalensis* (MIC 0.8 mg/ml) and *E. abyssinica* (MIC 0.8 mg/ml) extracts against *Vibrio cholerae* followed by the *L. schimperi* extract with an MIC of 1.6 mg/ml against *S. typhi*. The *L. schimperi* extract was inactive against *E. coli* and *V. choleraee*, while the *E. abyssinica* extract was inactive against *K. pneumoniae*. When the extracts of the five plants were combined (C1) they exhibited antimicrobial activity against all the four bacteria with MICs between 0.8 – 12.5 mg/ml (Table 4). The combination gave MIC values of 0.8 mg/ml against *S. typhi* and *E. coli* and 1.6 mg/ml against *K. pneumoniae* while the MIC for *V. choleraee* was 12.5 mg/ml. Similarly the other combinations C2 (*Rynchosia resinosa* + *Maytenus senegalensis* + *Entada abyssinica*), C3 (*Lannea schimperi* + *Rynchosia recinosa* + *Maytenus senegalensis)*, and C4 (*Lannea schimperi* + *Rynchosia resinosa* + *Ozoroa insignis*) that were tested (Table 4) were more active than the individual plant extracts.

**Table 3:** Results for antibacterial activity of the individual plant extracts

**Test organisms**	**Plant extracts (MICs mg/ml)**					
	***L.shimperi***	***R.recinosa***	***O.insignis***	***M.senegalensis***	***E.abyssinica***	**Gentamicin sulphate**
***K.pneumoniae***	6.25	12.5	NA	12.5	NA	0.000325
***S.typhi***	1.6	6.25	NA	6.25	12.5	0.00125
***E.coli***	NA	6.25	NA	12.5	12.5	0.000625
***V.cholerae***	NA	6.25	NA	0.8	0.8	0.00156

Key: NA = MIC > 12.5 mg/ml.

**Table 4:** Antibacterial activity of the combined plant extracts

**Test organisms**	**MICs (mg/ml)**						
	**C1**	**C2**	**C3**	**C4**	**C5**	**C6**	**Gentamicin sulphate**
***K.pneumoniae***	1.6	0.8	3.12	6.25	1.6	6.25	0.000325
***S.typhi***	0.8	0.8	0.8	1.6	0.8	6.25	0.00125
***E.coli***	0.8	1.6	0.8	1.6	1.6	6.25	0.000625
***V.cholerae***	12.5	12.5	6.25	6.25	12.5	6.25	0.00156

**Key:** C1 = *Rynchosia resinosa* + *Ozoroa insignis* + *Maytenus senegalensis* + *Entada abyssinica* + *Lannea schimperi*; C2 = *Rynchosia resinosa* + *Maytenus senegalensis* + *Entada abyssinica;* C3 = *Rynchosia recinosa* + *Lannea schimperi* + *Maytenus senegalensis*; C4 = *Rynchosia resinosa* + *Lannea schimperi* + *Ozoroa insignis;* C5 *= Rynchosia resinosa* + *Entada abyssinica* + *Lannea schimperi*; C6 = *Rynchosia resinosa* + *Maytenus senegalensis* + *Lannea schimperi*.

### Brine shrimp toxicity test (BST)

Table 5 shows that the *Ozoroa insignis* extract (LC_50_ =10.63 μg/ml) was the most toxic to the brine shrimp larvae. The extract of *Maytenus senegalensis* was the next toxic (LC_50_ =81.63 μg/ml) but the other plant extracts were virtually non-toxic with LC_50_ values ranging from 128.41-222.43 μg/ml. The combined ethanol extracts of the five plants gave an LC_50_ value of 66.12μg/ml.

**Table 5:** Results for brine shrimp toxicity of plant extracts

**Extracts**	**LC**_**50**_**(μg/ml)**	**95% CI (μg/ml)**
*Entada abyssinica*	140.39	102.47-192.34
*Lannea shimperi*	128.41	100.32-164.40
*Rhynchosia recinosa*	222.43	151.31-326.97
*Ozoroa insignis*	10.63	7.65-14.78
*Maytenus senegalensis*	81.63	64.79-102.84
Combined extract of 5 plants	66.12	52.52-83.24
Cyclophosphamide	16.30	12.01–22.30

### Acute toxicity in mice

The 80% ethanol extract of *Ozoroa insignis* was toxic to mice at doses above 1000 mg/kg body wt; causing hyperventilation within 30 min of extract administration, increased defaecation and passage of loose stools. The mice showed scruffy hair mostly after 24–72 h. Two mice died and 4 exhibited diarrhea at the dose of 2000 mg/kg body wt. Mice fed the *Entada abyssinica* extract did not show any toxicity up to 2000 mg/kg body wt but above this dose the mice exhibited increased respiratory rate and scruffy hair. Two mice died at the dose of 3000 mg/kg body wt. The *Lannea schimperi* extract caused increased defaecation/diarrhea but it did not kill any mice up to 2000 mg/kg body wt. Mortality to mice occurred at doses of 3000 mg/kg body wt and above. *Maytenus senegalensis* and *Rhynchosia resinosa* extracts were well tolerated by the mice up to 5000 mg/kg body wt. Unlike the single plant extracts, the combined extract was well tolerated by the mice up to 5000 mg/kg body wt, showing no signs of toxicity or death.

## Discussion

Ethanol induced gastric ulceration is due to a number of mechanisms that include depletion of gastric mucus and breaking the mucosal barrier, back diffusion of acid, increased gastric mucosal permeability, leading to increased leakage of hydrogen ions from the lumen, and decreased transluminal electrical potential difference [18]. It also causes changes in mucosal blood flow, destruction of microvascular and nonvascular cells, mast cell degranulation, neutrophil mediated mucosal injury [18], and depletion of certain oxygen free radical scavengers [18]. Metabolism of ethanol also releases free radicals [19]. The proton pump inhibitor pantoprazole protected the rat gastric mucosa from insult by acidified ethanol suggesting role of gastric acid in the ethanol/HCl-induced gastric ulceration. There was a clear change in the gross appearance of the gastric mucosa as compared to the solvent treated group. The polyherbal extract, at the dose of 800 mg/kg body wt reduced the degree of gastric mucosal ulceration with an efficacy that was comparable to that of 40 mg/kg body wt pantoprazole. Phytochemical screening indicated that the polyherbal extract contained tannins and flavonoids, which are phenolic compounds. Plant phenolics are known to be antioxidants and free radical scavengers [20]. Terpenoids which have antioxidant activity were also present in the extract. Since ethanol-induced gastric ulceration causes neutrophil mediated mucosal injury through release of oxygen free radicals, proteases and lysosomal enzymes [19], the phytochemical results may suggest that the extract owe its anti-ulcerogenic effect partly due to the presence of these compounds which have antioxidant and free radical scavenging properties. A recent report indicates that a methanol extract of *Entada abyssinica* stem bark exhibited free radical scavenging activity and it was suggested that phenols, flavonoids, saponins and tannins identified in the extract and fractions were responsible for their antioxidant activity [21]. In the current results saponins were not detected in the *E. abyssinica* ethanol extract. Furthermore *Entada abyssinica* is reported to have anti-inflammatory activity and it inhibited acetic acid-induced vascular permeability in a dose-dependent manner in mice [22]. Inflammation is implicated in acidified ethanol-induced gastric ulceration [19]. This may explain the rationale for including *E. abyssinica* in the polyherbal preparation by traditional healers.

Some plants of the genus Maytenus are popular in Brazil as traditional antiulcer and anti-inflammatory remedies [23,24]. Some of the species that already have proven antiulcer activity include *Maytenus ilicifolia*[18], *Maytenus robusta*[24], *Maytenus obtusifolia*[25], *Maytenus aquifolium*[26], and *Maytenus truncata*[27]. The hyroalcoholic extract of the aerial parts of *Maytenus robusta*, for example, reduced lesion index in a dose-dependent manner reaching maximum gastro-protection of 85.0 ± 9.2% at a dose of 500 mg/kg body wt as compared to 30 mg/kg body wt omeprazole which conferred 86.6 ±7.4% protection. The extract reduced the volume of gastric juice, total acidity and increased gastric pH [26]. These results are very similar to the results of this study and suggestion can be made here that the rationale for *Maytenus senegalensis* in this traditional antiulcer remedy is related to its potential to directly intercept the ulcerogenic mechanisms. Although it was not planned to measure volume of gastric juice in this study it was clearly observed that the stomachs of rats that were given the polyherbal extract had very little fluids, and in some cases, the stomachs had no fluids as opposed to the control ones from which we could draw up to 2 ml of gastric juice. Plants of this genus are also reported to have anti-inflammatory [23] and antioxidant activity [28], and these two processes are implicated in the mechanisms of acidified ethanol-induced gastric ulceration.

The phytochemical tests showed that *Lannea schimperi*, *Ozoroa insignis* and *Rhynchosia recinosa* also contained terpenoids and phenolics which are also likely to contribute to the anti-ulcerogenic activity of the polyherbal mixture.

In terms of meaningful clinical application the extract dose of 800 mg/kg boy wt is too large, so one can only speculate that the active components of the extract are compounds present in much lower concentrations. These compounds may be antioxidants and free radical scavengers, but given the fact that pantoprazole was equally effective these results still do not provide conclusive evidence for deducing the responsible anti-ulcerogenic mechanisms of the extract. More gastric ulcer models are, therefore, needed to determine the mechanisms of action of the extract and/or the active compound/s.

The most appropriate bacterium for this study was *Helicobacter pylori*, as this is the bacterium that is associated with peptic ulcer disease. Unavailability of this organism necessitated the use of other Gram negative bacteria; therefore the results reported in this study are only indicative rather than intended to imply possible anti-*Helicobacter pylori* activity. It is noted, however, that the individual plant extracts were not very active against the four Gram negative bacteria used but when their extracts were combined (C1) as used by traditional healers they showed higher activity. The different combinations were tested as an attempt to determine which combination would give the best antibacterial activity and probably determine the most important plant for use with *R. recinosa*. The results did not give an explicit clue on which plant is best for antibacterial activity when combined with *R. recinosa*. These results, however, show that the combination as used by traditional healers in which *R. recinosa* is used in combination with the other four plants is probably the best combination to achieve safety and efficacy.

Using the criteria suggested by Algiannis and colleagues [29], MICs up to 0.5 mg/ml represent strong activity; MICs between 0.6 and 1.5 mg/ml moderate activity, and MICs above 1.6 mg/ml weak activity. Based on these criteria it is evident that the combined extract exhibited weak to moderate antibacterial activity. These results support the observation reported earlier that multi-plant extracts are superior over single plant extracts and could be developed into more potent antimicrobials against resistant pathogens [30]. However, the antibacterial activity of the extracts was weak so unless actual testing is done using *Helicobacter pylori* it will be unrealistic to make any speculations regarding clinical efficacy.

This is not the first study to report the antibacterial activity of *Entada abyssinica* or *Ozoroa insignis*. *Entada abyssinica* has been reported to have antibacterial activity against *Klebsiella pneumoniae*, *Salmonella typhi*, *Escherichia coli*, *Pseudomonas aeruginosa* and other organisms [21]. *Ozoroa insignis* has also been reported to have antibacterial activity against Gram positive and Gram negative bacteria which include all the bacteria used in this study [31]. Similarly *Maytenus senegalensis* is reported to have antibacterial activity and the antibacterial compound, 3-oxo-friedelan-20α-oic acid, has been isolated from the root bark [32]. However, it is the first time the antibacterial activity of *Rhynchosia recinosa* and *Lannea schimperi* is being reported. The results (Table 3) show that the combinations C5 and C6 containing *R.recinosa* and *L. schimperi* with *E.abyssinica* and *M.senegalensis*, respectively were the least active. In fact C6 was less active than C5. It is evident that the reported results are inconclusive, and it is prudent that when *Helicobacter pylori* become available the effects of the extracts should be re-investigated.

Two preliminary explorative studies were done to investigate acute toxic effects of the plant extracts; acute toxicity in mice and the brine shrimp lethality test. *Maytenus senegalensis* and *Rhynchosia recinosa* extracts were well tolerated by mice up to 5000 mg/kg body wt. The ethanol extract of *Maytenus obtusifolia* which belongs to the same genus was earlier reported to exhibit low toxicity against brine shrimps and mice [25]. Information from the literature indicate that *E. abyssinica* has weak cytotoxic activity against some human cell lines, including human colon cancer cells [33] and is used traditionally for treatment of cancer else where in Tanzania [33]. However, both the brine shrimp results and acute toxicity in mice indicated a good safety margin of up to 2000 mg/kg body wt. The *Lannea schimperi* extract was also equally innocuous on acute administration. The *Ozoroa insignis* extract was the most toxic to both mice and brine shrimps. The literature indicate that *Ozoroa insignis* stem bark and root methanol extracts were cytotoxic against different human cell lines including liver, bladder and mammary cancer cell lines [34,35] and has topoisomerase II inhibitory activity [36]. Despite the seemingly toxic profile of, in particular *Ozoroa insignis*, the polyherbal formulation showed very low acute toxicity in mice and in the brine shrimp lethality test. The combination of extracts of the five plant extracts was well tolerated during the whole 14 day observation period and at doses up to 5000 mg/kg body wt.

## Conclusion

The combined extract of the five plants exhibited a dose-dependent cytoprotective activity in the rat ethanol-HCl gastric ulcer model. The extracts also exhibited weak antibacterial activity against four Gram negative bacteria and low acute toxicity in mice and brine shrimps. Although the results seem to support claims by traditional healers who use a decoction of the five plants for treatment of peptic ulcers, more models of gastric ulceration and proper animal toxicity studies are needed to validate possible clinical use of the polyherbal extract.

## Competing interests

The authors have no competing interests for this research, and share the aspirations of the traditional healers of the Kagera Region to bring good healthcare services to their community.

## Authors’ contributions

EEH did the whole work, including preparation of the plant material, extraction, bioassays etc. under the guidance of RSON who worked closely with him in the laboratory. MJM did the ethnomedical studies that documented these plants and identified the plants for this study; participated in advising design of the study and provided the theoretical guidance of the project. MJM also drafted this manuscript including all the revisions. RSON participated in the experiments and was the laboratory supervisor for the study. He also participated in designing the study. DTM did phytochemical screening of the extracts. RLAM is a co-mentor of EEH and participated in advising design of the study and proposal development. All authors read and approved the final manuscript.
